# Management Approach of Pediatric Constipation

**DOI:** 10.7759/cureus.19157

**Published:** 2021-10-31

**Authors:** Abdulrahman A Alnaim

**Affiliations:** 1 Pediatrics, King Faisal University, Alhasa, SAU

**Keywords:** pediatric, constipation, management, approach, causes

## Abstract

Constipation is a common challenge in pediatrics. Abdominal radiographs are frequently taken in the pediatric emergency department for diagnosis despite their inadequate reliability to detect the pathology or the degree of constipation. Misdiagnosis of constipation may cause multiple vague physician visits, deployment of emergency medical services, use of radiation, unnecessary laboratory tests, and even surgical procedures. The primary evidence-based suggestions are based on published guidelines that include management of constipation in children divided into three stages of therapy: (1) disimpaction, (2) maintenance therapy, and (3) behavior modification, and special care should be given to neonates and to children with pre-existing medical problems.

## Introduction and background

Constipation is considered a symptom, instead of disease [1]. It is identified as infrequent motions or the passing of hard stools. Constipation may be considered if less than three bowel motions per week. About 0.5% of school children defecate less than three times per week and 0.3% have incontinence in defecation [2]. Additionally, 20% of children have been reported to have at least one clinical feature of constipation. Thus, it is essential to apply diagnostic criteria to identify constipation [1]. Bowel movement in children differs from adults. Children’s bowel habits have a wide normal spectrum [3]. In general, an infant’s daily bowel movements are about 3-4, a toddler’s daily bowel movements about 2-3, and at age 4, children’s daily bowel movements develop as an adult about 1-2 stools [4]. Less than three bowel movements per week, painful or retention stools, with or without encopresis are considered as constipation [5].

Emergency care is required more by children with constipation than children without constipation [6]. Children who seek emergency care due to symptoms secondary to constipation mostly have fecal impaction needing disimpaction followed by maintenance therapy [7]. A limited number of studies showed that disimpaction is effective, which is done using oral or rectal drugs or a mix of them [8]. The oral approach is less invasive, however, adherence is problematic. The rectal approach is faster, however, it is more invasive [9]. Polyethylene glycol (PEG)-based laxatives are effective and safe to be used for the treatment of chronic constipation in outpatient care [10], however, research about their use during emergencies is limited [9]. In addition, a previous clinical trial found that disimpaction using enema helps immediate relief of symptoms when compared to PEG [9]. However, enema use can cause some side effects such as becoming upset [9] and electrolyte disturbance when using sodium phosphate enemas such as hypocalcemia, hypokalemia, hyperphosphatemia, and hypernatremia [11], which can lead to severe neurologic disturbance and death, although this is infrequent [12]. 

A previous study showed that children coming to the emergency room having constipation revisited it especially due to vomiting, enema administration, diagnostic imaging, and significant medical history [13]. So, this review aimed to find the best evidence management of constipation in children.

## Review

Etiology

Constipation can be divided into a primary cause or secondary cause due to other factors. Thus, constipation is divided to two main types: organic (5%) and functional (95%) types [14].

Organic Causes

Organic causes may be due to specific structural abnormalities as anal stenosis, anteriorly displaced anus, and imperforate anus. Organic causes can also be due to neurological causes as myelomeningocele, occult spina bifida, tethered cord, spinal cord tumor, or infection. This is because constipation may happen due to abnormal neurologic innervation, as a result of weak muscle tone contraction of the anal sphincter leading to the closure of the anal canal rather than relaxation during try to defecate) [15].

Constipation can be also due to metabolic causes such as diabetes insipidus, hypercalcemia, hypokalemia, or hypothyroidism. Drugs as anticonvulsants, antipsychotic or codeine-containing anti-diarrheal can also cause constipation. Other organic causes include intestinal disorders as celiac disease (gluten enteropathy), cow’s milk protein intolerance (milk protein allergy), cystic fibrosis, Hirschsprung disease, irritable bowel syndrome [14].

Hirschsprung disease is the most important organic cause, named congenital aganglionic megacolon, which can cause toxic megacolon if a diagnosis was missed. It is due to deficiency or absence of ganglion cells in the colon, causing tonic contraction of a segment of the bowel. Symptoms commonly appear at birth with a lag in meconium passage. Nevertheless, it appears in childhood, just when a short segment of the bowel is involved. It is more common in males than females (4:1) and in children with Down syndrome [5].

Functional Causes

Functional causes are defined as the difficulty of passing stools for causes that differ from organic causes. It may be due to low fiber, decrease toilet stress, or desire to control [16]. Functional constipation is divided into three subtypes: constipation with slow-transit, normal transit, and rectal evacuation disorders. Feces are pushed through the colon by intestinal contractions. About 13-25% of children have slow-transit constipation [17].

Functional constipation is believed to be caused by multiple factors. These factors involve genetic factors, lifestyle factors such as diet during the infancy period, feeding changes, as the switch from breastfeeding to formula feeding or the initiation of solid foods, usually trigger functional constipation [18]. However, an association of cow’s milk protein allergy with functional constipation is a debatable matter, and constipation is occasionally thought to be caused by food allergy. Histological changes of the children's colonic mucosa with chronic constipation suggest the presence of inflammation [19]. Low fiber and fluids intake cause constipation especially in school children, adolescents, and adults [20]. Moreover, eating disorders should be considered in adolescents with constipation [21]. For example, in patients suffering from anorexia nervosa or bulimia, constipation occurs. However, obesity and physical activity are controversial matters as it is thought that mechanical effects of obesity produce tension on the pelvic floor [22], and so does low physical activity.

Colonic motility dysfunction is believed to cause constipation with delayed colonic transit time. That’s proved by colonic manometry studies, which state that high-amplitude propagating contractions (HAPCs) are less common in patients who are suffering from slow-transit constipation than in those not suffering from constipation [23]. HAPCs are believed to help colonic content movement in anterograde movement. This usually occurs after awakening and eating. It is thought that retrograde cyclic motor pattern occurs in children with functional constipation but less frequently [24]. These contractions propagate in a retrograde direction. The clinical value of these findings is still uncertain. Impairment of anorectal function or structure causes difficulty in ejecting stools from the rectum, which are usually related to symptoms of straining and a sensation of incomplete evacuation. Dyssynergia defecation is the most common one [25].

Psychological factors, such as patients with behavioral disorders like autism and attention deficit hyperactive syndrome, have been reported to cause functional constipation [26]. Stressful life events as physical or psychological trauma may cause functional constipation because these events occur more commonly in patients with functional constipation [27]. Sensations in children having functional constipation as abdominal distention and pain are handled from the colon to the cerebral cortex through afferent nerves via the enteric nervous system. These may affect brain handling leading to psychological disorders. It is thought that emotional and psychological mechanisms could modify colonic function through efferent pathways, causing gastrointestinal dysfunction [28]. This theory can be supported by MRI-based studies which reported that in comparison with healthy people, patients having functional constipation revealed different forms of brain handling reaction to distention of rectum and a distinctive control brain activity [29]. 

Behavioral factors also can cause functional constipation. The commonest cause of childhood constipation is withholding behavior. Stool withholding behavior could be prompted by having previous painful or hard stool which is worsened by an anal fissures history or anxiety of toileting. Stool withholding behavior causes harder stools as water is absorbed by the colonic mucosa, causing more painful defecation which is a vicious cycle [30].

Evaluation

History

Medical history and complete physical examination can confirm the diagnosis. Detection of alarm symptoms differentiates the underlying organic conditions [31]. Alarm symptoms or signs in children differ according to the age of onset of symptoms which include a history of delayed meconium passage, onset before one month old, family history of Hirschsprung disease, hypothyroidism or coeliac disease, bloody stool, fever, bilious vomiting, or ribbon stool.

Physical Exam

Alarm signs also include physical examination (including digital rectal examination) that detects failure to thrive, severe abdominal distention, tight and empty rectum, abnormal cremasteric or anal reflex, abnormal gluteal cleft or position of the anus, anal scars or fissures, major fear of per rectal exam, or anal hematoma. A neurological exam that is abnormal also can reveal alarm signs such as lack of lumbo-sacral curve, sacral dimple, spinal hair tuft, thyroid gland abnormalities, eczema, a gush of liquid stool, and air on withdrawal of finger [30].

Diagnostic Studies

Rome IV criteria are used for clinical diagnosis of functional constipation. The symptoms of functional constipation are based on Rome's criteria. The first Rome criteria for pediatric gastrointestinal functional problems was the Rome II which was published in 1999. Moreover, the modified one was Rome IV that was published in 2016 as criteria for childhood and adult functional constipation [32].

Rome IV criteria for functional constipation says that the patient should have two or more of the criteria (Table 1) for one month or more. The criteria for infants and toddlers are shown in Table 1 [33]. For children and adolescents, a patient should not meet the suggested criteria for irritable bowel syndrome (IBS) [33].

**Table 1:** Rome IV criteria for infants and toddlers

Criterion
Less than or equal to two defecations per week
History of painful or hard bowel movements
History of extreme stool retention
History of large thickness stools
Presence of a large fecal mass in the rectum
One or more episodes of fecal incontinence per week
History of large thickness stools that can end the toilet

Symptoms of the most common type of constipation in children (functional constipation) involve hard stool, infrequent bowel movements, usually is associated with symptoms of bloating and abdominal pain, and fecal incontinence which is involuntary loss of stools in the underwear due to overflow of soft stool around solid feces in the rectum [2]. It may be associated with urinary symptoms, like urinary incontinence and urinary tract infections [34].

In addition to history and physical examination, there are other investigations. They are suggested only in the existence of alarm symptoms or failure of conventional therapeutic approaches. It is used in adults more than in children. It is applied to exclude organic constipation and to differentiate between types of functional constipation (slow-transit, normal-transit, or rectal evacuation disorder) [35]. The most commonly used tests including laboratory testing (complete blood count, thyroid function tests, biochemical profile, and coeliac disease investigations) if alarm symptoms occur in a child having marked weight loss or short stature, chronic gastrointestinal symptoms, or family history that should consider serological testing for coeliac disease and thyroid function [30]. Routine X-ray use for constipation diagnosis is not advised in children [31].

Studies presented that calculating colonic transit time (CTT) is effective in differentiation between a normal and abnormal colonic motor function with severe constipation. Delay in CTT shows slow-transit constipation. However, withholding behavior in children also can cause (segmental) colonic slow transit [36]. Anorectal manometry in children is used for excluding Hirschsprung disease through measuring the existence of inhibitory recto-anal reflex [31]. Nevertheless, children with a high suspicion of Hirschsprung disease are preferred to do the rectal biopsy. Colonic manometry is performed in an academic setting for children which is useful with difficult constipation for excluding neuromuscular motility abnormalities of the colon combined with slow-transit constipation [37]. Contrast enema is helpful for excluding structural abnormalities such as masses, megarectum, and megacolon in children. it can be useful in children with severe symptoms as it is used as a guide in surgical interventions via giving information about colonic anatomy, length, and dilatation [38].

The diagnostic testing summary is shown in Table 2 [14].

**Table 2:** Diagnostic testing summary

Disease/condition	Diagnostic testing
Hirschsprung disease	Barium enema, rectal manometry, and biopsy.
Tethered spinal cord or tumor	Plain x-rays of lumbosacral spine and MRI.
Hypothyroidism	Thyroid-stimulating hormone and thyroxine level.
Lead poisoning	Blood lead level.
Infant botulism	Stool analysis for botulinum toxin.
Cystic fibrosis	Sweat test and genetic testing.
Metabolic derangement	Calcium and other electrolytes levels.
Celiac disease	Serologic screening is usually for IgA antibodies to tissue transglutaminase.

Treatment

The first step in the management of functional constipation is nonpharmacological treatment including health education and lifestyle modification as a healthy diet, regular exercise, and education about proper toileting posture [31].

Non-pharmacological Treatment

Dietary recommendations, which are the first interventions, to take a normal fiber and fluid can treat constipation [31]. Fibers are recommended as 0.5 g/kg daily in children more than 5 years old [39].

Program training to toilet with rewards and educating children to defecate two times at least per day help prevention of fecal impaction which decreases the risk for fecal incontinence [30].

Pharmacological Interventions

Pharmacological treatment of functional constipation in children includes two steps, disimpaction then maintenance therapy. Disimpaction by high-dose oral polyethylene glycol (PEG) or active components enemas as sodium docusate, sodium lauryl sulfoacetate, or sodium phosphate. However, high-dose PEG may increase the risk of fecal incontinence [31]. After successful disimpaction, maintenance therapy should be done to avoid the repeated collection of stools. The first choice for functional constipation is osmotic laxatives [31]. PEG is considered the first-choice osmotic mediator due to its effectiveness and safety [40].

New Pharmacological Mediators

Prosecretory drugs as linaclotide, plecanatide, and lubiprostone adjust intestinal epithelial channels, stimulating intestinal fluids secretion and improving stool volume, causing improvement of gastrointestinal transit [41]. Lubiprostone stimulates the secretion of intestinal fluid via stimulating the cystic fibrosis transmembrane conductance regulator and CIC2 chloride channel, however, this needs more studies as only one study done on the pediatric was published. Lubiprostone was found to be safe and improved secondary outcomes as pain, stool consistency, and straining during defecation [42].

Acetylcholinesterase inhibitors as pyridostigmine surge gastrointestinal motility via increasing acetylcholine bioavailability. Only one paper about pyridostigmine comprising four children having gut motility disorders proposed a valuable effect on defecation frequency only in one child having chronic constipation [43].

Trans-anal Irrigation

It is usually used in children with functional constipation whose pharmacological treatment failure. Trans-anal colonic irrigation is recognized to be used in patients with neurogenic bowel disorders and anorectal malformations [44]. In some studies, it was found to be effective for the treatment of fecal incontinence and symptoms of constipation [45].

Surgical Interventions

Surgical approaches vary broadly between physicians especially when therapies fail, and symptoms are seriously interrupting the quality of life [31].

Antegrade continence enemas (ACEs): ACEs allow the washing out of fluids with or without laxatives via an external opening into the colonic lumen. ACE surgery is a minimally invasive surgery with excellent clinical outcomes in children [46].

Ostomies and resection: In case of failure of minimally invasive surgical therapies (problematic functional constipation), ileostomy, colostomy, or total colectomy can be used [47]. The choice of type of surgery depends on the existence of colonic dilation and dysmotility according to contrast enema and manometry results. It can be effective in children with megarectum or mega-sigmoid [48]. Total or subtotal colectomy with or without ACE can be of benefit in children with colonic dysmotility or slow-transit constipation [49].

Neuromodulation

It includes abdominal transcutaneous electrical stimulation, percutaneous tibial nerve stimulation, and sacral nerve stimulation. Sacral nerve stimulation, by stimulating S3 and S4, showed effectiveness on both urinary and fecal incontinence [50], but its mechanism of action is unclear [51]. Follow-up for a long-term period in children having heterogeneous causes of constipation caused non statistically significant frequency improvement of defecation after two years. Nevertheless, fecal incontinence rates were reduced from 72% to 20% [52]. Abdominal transcutaneous electrical stimulation and percutaneous tibial nerve stimulation utilize noninvasive or minimally invasive methods. Long-term follow-up showed improvement of consistency and fecal incontinence in 33% of children with slow-transit constipation after 2 years [53].

Novel alternative therapies involving acupuncture and fecal microbiota have also been used [30].

Management During Emergencies

A previous study by Beinvogl et al. found that the causes of getting an abdominal radiograph were for evaluation of stool burden, fecal impaction, to know the cause of abdominal pain, and assessment of treatment response. The management plan was changed based on the radiographic findings and confidence in the management plan was increased based on it, although most studies reported that imaging findings poorly correlate with severity [54].

Another study reported that abdominal imaging in the emergency department can be avoided when a digital rectal exam (DRE) is done. It reported also increasing hospital admission for children with abdominal pain who had abdominal imaging [55].

Functional constipation is a highly common condition among children that causes many emergency department visits. Physicians in the emergency department should know its different presentations and that it may be misdiagnosed with different serious conditions as intussusception and appendicitis. In addition, they should consider organic causes to perform investigations urgently. They should be aware of the current treatment modalities and of the need for disimpaction before maintenance therapy for successful treatment [7].

## Conclusions

Research has proved that some laboratory testing or abdominal X-rays are unwarranted for diagnosing. At least 2 months of combination between education, toilet training, and an early laxative therapy is effective in the majority of children with functional constipation. Currently, studies on the use of prebiotics, probiotics, and synbiotics are not enough. New drugs as lubiprostone, prucalopride, and linaclotide have not yet been evaluated on large randomized controlled studies in children with functional constipation, despite showing effectiveness in adults.

Currently, new non-pharmacological treatments as neuromodulation have shown assurance but need to be evaluated on large randomized controlled studies in children with functional constipation. Surgery is the last choice in the treatment of children with failure of all therapies. The benefits of surgical interventions must be concerned against the risks of complications and stoma problems.

Children attending to the emergency department with abdominal pain and getting therapy for fecal impaction can be discharged with advice for preventive therapy for constipation. Some pediatric patients need hospital admission for further investigation or as patients with organic causes of constipation as Hirschsprung disease, structural disorder, or neurological causes.
